# Knowledge and experience of pediatricians and pedodontists in identifying and managing child abuse and neglect cases

**DOI:** 10.1097/MD.0000000000037548

**Published:** 2024-03-22

**Authors:** Hazal Özer, Merve Abakli İnci

**Affiliations:** a Department of Pediatric Dentistry, Faculty of Dentistry, Necmettin Erbakan University, Konya, Turkey; b Department of Pediatric Dentistry, Faculty of Dentistry, Necmettin Erbakan University, Konya, Turkey.

**Keywords:** child abuse, child neglect, pediatrician, pedodontist

## Abstract

Child abuse, by definition parents and other family members, caregivers, or any adult he does not know culturally inappropriate, harmful to the child described, inhibiting growth and development, or exposure to a restrictive behavior. This study aims to evaluate the capabilities of pediatricians and pedodontists in identifying and managing child abuse and neglect within healthcare settings, a crucial responsibility for professionals across various medical disciplines. Questionnaire was performed on 53 pediatricians and 89 pedodontists. Utilizing a 28-item expert-designed electronic questionnaire, the study solicited responses from pediatricians and pedodontists to assess their demographic characteristics, professional experience, and self-perceived competence in this critical area. The results indicate that 42% of the participating healthcare providers have encountered at least one case of child abuse and neglect. Notably, pedodontists displayed a higher level of uncertainty in identifying abuse and neglect cases compared to pediatricians. Furthermore, participants who had a higher level of self-perceived competence were significantly more willing to identify and manage cases, although this self-assessment did not correlate with their actual skills or level of willingness to intervene effectively. The study concludes that there is a pressing need for specialized training programs tailored to enhance the skill sets of healthcare providers in identifying and managing child abuse and neglect. These programs should encompass not only academic knowledge but also practical applications and psychosocial support techniques to ensure a holistic approach to combating this serious issue.

## 1. Introduction

Parenting requires constant attention and sensitivity. It is imperative to provide children with consistent support, protection, and guidance. In addition to parents, who must have the necessary skills to prevent child abuse under any circumstances, other elements of society must also do their part with the understanding that securing the well-being and future of children is a collective responsibility. In this context, the role of healthcare professionals is to ensure that the family receives appropriate care and accurate information before and after birth to ensure children’s future health and well-being. The foundations of a child’s sense of trust are laid by meeting children’s basic needs, including nutritional, sleep, and hygiene needs, in an orderly and appropriate manner during infancy.^[1]^

Child abuse is the exposure of a child to behaviors by parents and/or other family members, caregivers, or unfamiliar adults that are culturally inappropriate, harmful to the child, or inhibit growth and development.^[2]^ Sexual and physical abuse of children was uttered for the first time by Tardieu at the Paris Medical Academy in 1860. Later, Caffey in 1946 and Kemple in 1961 drew attention to the importance of this concept through the Battered Child Syndrome.^[3]^ Child abuse is defined by the World Health Organization (WHO) as any adult behavior that negatively affects the health, physical, and psychosocial development of a child under the age of 18.^[4]^ On the other hand, “child neglect” is defined as the omission of meeting a child’s basic needs, including nutrition, clothing, shelter, education, health, and affection by biological parents or legal guardians and other caregivers, preventing the child’s physical, emotional, moral or social development.^[5]^

Child abuse may have an acute impact on children’s physical and mental health and/or may give rise to serious health problems at later ages, including developmental disorders, sleep disorders, eating disorders, alcohol or drug addiction, depression, anxiety, panic disorder, increased delinquency, violent behaviors, self-harm, suicidal tendencies and abusive behaviors.^[6,7]^

WHO reported that approximately 53,000 children worldwide died from abuse or neglect in 2002.^[8]^ According to a study conducted by the United Nations Children’s Fund and the Ministry of Labor and Social Security of the Republic of Turkey on children aged 7 to 18 years, 45% of the children were exposed to physical abuse, 50% were ex-posed to emotional abuse and 25% were neglected.^[9]^In addition, a retrospective study investigating 523 pediatric cases for whom a forensic report due to abuse was issued be-tween 2014 and 2019 in Turkey determined that 39.8% of the cases were victims of sexual abuse.^[10]^

A study conducted on primary care physicians found that 64.3% of the physicians were willing to report child abuse cases, and 62% felt that they had sufficient knowledge to manage child abuse cases.^[11]^ In the same study, the rate of physicians who thought that child abuse cases were underreported was 64%. A questionnaire study revealed that only 53% of the answers given by family physicians and pediatricians to questions about child abuse were correct. It was determined that the problems faced by doctors in managing child abuse cases were primarily due to the lack of education on this subject, emotion-al difficulties, and perceiving abuse as a social problem rather than a medical problem.^[12]^However, the task of identifying and managing child abuse and neglect cases among healthcare professionals should not be the sole responsibility of pediatricians and general practitioners among healthcare professionals. Healthcare professionals from other disciplines should also contribute equally to this task. Studies have shown that among healthcare professionals from different disciplines, the rate of identifying and managing child abuse and neglect cases is low, especially among dentists. The main barriers to re-porting child abuse and neglect are lack of history of child abuse and neglect or lack of access to existing history of child abuse and neglect, lack of awareness about child abuse, and the problems that can be experienced in relation to legal procedures in case of reporting child abuse.^[13,14]^

Child abuse and neglect remain major public health problems worldwide. Although progress has been made in this field through training and awareness-raising activities, public policy and legislation, family support programs, and healthcare and social service programs, there is still a long way to go. Training and awareness-raising activities are among the most essential tools in combating child abuse and neglect. All healthcare professionals who come into contact with children in the course of their work are targeted with these training and awareness-raising activities. After raising awareness, it is important to develop the skills necessary to identify and manage child abuse and neglect cases in healthcare professionals, both from medical and legal aspects. The next step in addressing child abuse and neglect cases from the point of view of healthcare professionals involves making the necessary adjustments and improvements to the process based on relevant experiences.^[10]^

Child abuse and neglect are prevalent, but most go unreported or underreported.^[15]^ Problems such as the perception that family problems, especially those related to cultural norms and values, are private, fear of the abused or neglected child or witnesses of the abuse or neglect from the perpetrator, or threats of the abused or neglected child or wit-nesses of the abuse or neglect by the perpetrator, lack of information on how to identify and report child abuse and neglect cases, the fear of the family that their children will be taken away, the indifference of professionals, and the inadequacy of the legislation pre-vent the reporting of child abuse and neglect cases.^[16]^

Given their patient population, pediatricians are most likely to encounter child abuse and neglect cases among healthcare professionals. However, healthcare professionals from other disciplines, including pedodontists, should also contribute equally to the task of identifying and managing child abuse and neglect cases.

In this context, this study was carried out to determine and compare pediatricians’ and pedodontists’ awareness of child abuse and neglect, their knowledge and skills in identifying child abuse and neglect cases, and their willingness/reluctance to report these cases, with a view determining the deficiencies of pediatricians and pedodontists in identifying and managing child abuse and neglect case.

## 2. Methods

### 2.1. Study design

The population of this observational study consisted of healthcare professionals aged be-tween 24 and 65 years who graduated from the faculties of medicine or dentistry and were actively involved in examining and treating pediatric patients as pediatricians and pedodontists at the time of the study. Pediatricians and pedodontists with no experience in emergency services or with less than 6 months of professional experience were excluded from the study. The study protocol was approved by the Ethics Committee for Drug and Non-Drug Product Research of the University Faculty of Dentistry (Approval No: 2020/02-13). The study was conducted in full compliance with the Declaration of Helsinki developed by the World Medical Association.

### 2.2. Data collection process

The link to the electronic questionnaire was sent to the participants’ e-mail addresses registered with the Turkish Pediatric Association and Turkish Society of Pediatric Dentistry or through social media and study groups in the event of pediatricians and pedodontists not registered with the said organizations in 2020. An electronic questionnaire was preferred because of its advantages, such as enabling reliable and accessible analysis, saving time, and fewer human errors.^[17]^ Each participant was provided with a questionnaire form containing questions about demographic characteristics, i.e., age and gender, professional status, specialization, institution, title and knowledge and skills regarding child abuse and neglect. The data collection process was completed over a period of 3 months.

### 2.3. Questionnaire development process

The questionnaire was developed in several stages. First, the medical conditions and behaviors observed in abused and neglected children were reviewed in the context of the study’s objectives to determine the relevant concepts. The items and statements to be included in the questionnaire were formulated based on these concepts, with a view to re-flecting different aspects of child abuse and neglect. After drafting the preliminary questionnaire, a pilot study was conducted to assess the time needed to complete the questionnaire, determine the statements that may be difficult to understand and address the necessary revisions. In the end, the questionnaire was finalized to consist of 28 questions in 3 sections assessing participants’ knowledge and skill levels in matters related to child abuse and neglect. Of these sections, the first section assessed participants’ level of knowledge and ability to identify cases of child abuse and neglect, the second section ad-dressed the difficulties faced by pediatricians and pedodontists in reporting cases of child abuse and neglect, and the final section discussed the importance of education in over-coming these challenges.

### 2.4. Assessment of content validity

The content validity of the questionnaire was assessed based on expert opinions. To this end, 3 academicians with expertise in the field were selected to provide feedback. The experts were given detailed information about the questionnaire’s content, items, and objectives. Subsequently, specific adjustments and additions were made to the questionnaire items based on the experts’ recommendations. In the end, the experts expressed their agreement that the questionnaire items accurately represented the concept being measured and were appropriately designed for the intended purpose.

### 2.5. Statistical analysis

The study sample consisted of 142 participants, of whom 53 were pediatricians, and 89 were pedodontists. The sample size was determined based on several factors, including the scope of the study, time constraints, and available resources. It is a general tendency to conduct a power analysis to determine sample size prior to the study. However, in our case, it was more practical to assess the adequacy of the sample size through a post hoc analysis due to unavoidable logistical considerations.

The post hoc power analysis was conducted assuming an alpha level of 0.05, in line with standard scientific research practices. We observed a medium-sized effect in our primary outcomes. Given these parameters, the post hoc power analysis indicated that the study achieved a statistical power of approximately 1. Although this exceeds the conventionally targeted statistical power of 0.8, it confirms the adequacy of the sample size for detecting medium-sized effects.

The diversity of our sample in terms of age, professional experience, and specialization of the participants adds a layer of robustness to our findings, enhancing their generalizability and, thereby, the study’s strength. In sum, even though a power analysis could not be made before the study due to logistical constraints, the post hoc power analysis confirmed that our study had enough power to detect medium-sized effects.

The descriptive statistics obtained from the collected data were tabulated using mean with standard deviation and median with minimum and maximum values in the case of continuous (numerical) variables depending on the normal distribution characteristics of the variables and summarized using number (n) and percentage (%) values in the case of categorical variables. Normal distribution characteristics of the continuous variables were analyzed by Shapiro–Wilk, Kolmogorov–Smirnov, and Anderson-Darling tests.

Two statistical tests were used to evaluate the differences in categorical variables between the groups. Accordingly, Pearson’s chi-square test was used in 2 × 2 tables where the expected number of cells was 5 and above, and Fisher’s exact test was used in tables where the expected number of cells was below 5.

Three different linear regression models were developed to assess healthcare professionals’ competencies and attitudes toward recognizing and reporting child abuse and neglect cases. Model 1 assessed healthcare professionals’ ability to recognize signs of child abuse and/or neglect; Model 2 assessed healthcare professionals’ ability to identify cases of child abuse and neglect (based on total scale scores); and Model 3 assessed their willingness to be involved in identifying and reporting cases of child abuse and neglect. Independent variables in each model included age, gender, specialization, institution, title, willingness to receive training on child abuse and neglect, and various attitudes and beliefs (e.g., fear of family/parental reaction in case of reporting child abuse and neglect, lack of knowledge about the protocol for reporting cases of child abuse and neglect, etc.).

The relationships between numerical variables, particularly the “Recognition Skills” scores and the scores regarding the items “To what extent can you recognize the signs and symptoms of child abuse and/or neglect?” and “To what extent do you intend to be involved in the process of identifying physical abuse of a child?” were analyzed. Spearman’s Rho correlation coefficient was used when the variables were not normally distributed.

Jamovi project 2.3.28 (Jamovi, version 2.3.28, 2023, retrieved from https://www.jamovi.org) and JASP 0.17.3.0 (Jeffreys’ Amazing Statistics Program, version 0.17.3.0, 2023, retrieved from https://jasp-stats.org) software packages were used to conduct the statistical analyses of the collected data. Probability (*P*) statistics of ≤ .05 were deemed to indicate statistical significance.

## 3. Results

Of the 142 participants included in the study sample, 107 were female, 35 (2.6%) were male, 53 (37.3%) were pediatricians, and 89 (62.7%) were pedodontists. Fifty-nine (41.5%) participants stated that they had previously encountered a case of child abuse and neglect, whereas 61 (43%) stated that they had never encountered a case of child abuse and neglect, and 22 (15.5%) stated that they encountered a case they suspected of child abuse and neglect. The rates of participants who stated to have encountered and not have encountered a case of child abuse and neglect were similar among participants aged 44 years and younger. On the other hand, it was determined that a higher rate of participants among participants over 44 stated that they encountered a case they suspected of child abuse and neglect compared to participants aged 44 years and younger (*P* < .001). The rates of participants who stated to have encountered a case of child abuse and neglect and a case they suspected of child abuse and neglect were similar (*P* > .05) yet significantly higher (*P* = .037) than the rate of participants who stated not to have encountered a case of child abuse and neglect among female participants. The rates of those who stated to have encountered and not to have encountered a case of child abuse and neglect were similar, yet lower than those who stated to have encountered a case they suspected of child abuse and neglect among pedodontists. On the other hand, the rate of those who stated to have encountered a case they suspected of child abuse and neglect was higher than the rates of those who stated to have encountered and not to have encountered a case of child abuse and neglect among pediatricians (*P* < .001) (Table 1).

**Table 1:** Demographic and professional characteristics of the participants.

	Overall (n = 142)	In your professional experience, have you seen any suspected cases of abuse and/or neglect among child patients?			*P* *
		No (n = 61)	Not sure (n = 22)	Yes (n = 59)	
Age group					
<44 age	134 (94.4)	58 (95.1) a	17 (77.3) b	59 (100.0) a	**<.001**
>44 yr	8 (5.6)	3 (4.9) a	5 (22.7) b	0 (0.0) a	
Gender					
Female	107 (75.4)	40 (65.6) a	20 (90.9) b	47 (79.7) a,b	**.037**
Male	35 (24.6)	21 (34.4) a	2 (9.1) b	12 (20.3) a,b	
Employment sector					
State Hospitals	123 (86.6)	54 (88.5)	18 (81.8)	51 (86.4)	.675
Private Hospitals/Medical Offices	19 (13.4)	7 (11.5)	4 (18.2)	8 (13.6)	
Branch					
Pediatrics	53 (37.3)	25 (41.0) a	1 (4.5) b	27 (45.8) a	**.002**
Pedodontics	89 (62.7)	36 (59.0) a	21 (95.5) b	32 (54.2) a	
Title					
Research staff	63 (44.4)	29 (47.5)	10 (45.5)	24 (40.7)	.397
Specialist	52 (36.6)	22 (36.1)	5 (22.7)	25 (42.4)	
Academic titles	27 (19.0)	10 (16.4)	7 (31.8)	10 (16.9)	

Bold values represent statistically significant results (*P* < 0.05).

Values are expressed as n (%).

a, b: Letters indicating significant differences between groups.

* Pearson Chi-Square or Fisher Freeman Halton test.

Sixty-three (44.4%) participants stated that they had either no or insufficient knowledge about how to deal with cases of child abuse and neglect, that there is a need for training on this subject, and that they would be willing to attend such training. The barriers to reporting child abuse and neglect were generally related to concerns about lack of knowledge and skills in identifying and managing cases of child abuse and neglect, misinformation about child abuse and neglect, and emotional reasons. The participants’ level of knowledge and skill in identifying cases of child abuse and neglect was 23/30, their perceived competence in recognizing and dealing with cases of abuse and neglect was 7/10, and their level of willingness to be involved in dealing with cases of abuse and neglect was 8/10 (Table 2).

**Table 2:** Descriptive statistics on participants’ knowledge and skill levels in recognizing child abuse cases, their perceptions of competence in recognizing and managing child abuse cases, and their willingness to be involved in abuse management processes.

		Overall (n = 142)
Total score of knowledge-skills for recognizing child abuse		23.0 [14.0–28.0]
To what extent do you feel competent to recognize and manage child abuse?		7.0 [3.0–10.0]
To what extent do you intend to be involved in child abuse management processes?		8.0 [1.0–10.0]
In your professional experience, have you encountered any suspected abuse and/or neglect among child patients?	No	61 (43.0)
	Not sure	22 (15.5)
	Yes	59 (41.5)
Physical abuse of children is one of the leading causes of child mortality.	No.	16 (11.3)
	Not sure	37 (26.1)
	Yes	89 (62.7)
Pedodontists are not as obliged as medical doctors to recognize cases of abuse.*	No.	10 (7.0)
	Not sure	17 (12.0)
	Yes	115 (81.0)
Bruises on the cheek can be caused by slapping or pinching the face with the fingers.	No.	0 (0)
	Not sure	6 (4.2)
	Yes	136 (95.8)
Recurrent dental trauma is unlikely to be caused by abuse.*	No.	14 (9.9)
	Not sure	21 (14.8)
	Yes	107 (75.4)
Abrasions and lacerations to the palate, vestibular region or floor of the mouth in infants may be signs of “force-feeding.”	No.	5 (3.5)
	Not sure	20 (14.1)
	Yes	117 (82.4)
Bruises around the neck are usually associated with accidental injuries.*	No.	29 (20.4)
	Not sure	23 (16.2)
	Yes	90 (63.4)
An abused child will usually tell someone immediately after the abuse.*	No.	11 (7.8)
	Not sure	16 (11.3)
	Yes	114 (80.9)
In most cases of abuse, the perpetrator is a someone the child does not know well.*	No.	111 (78.2)
	Not sure	19 (13.4)
	Yes	12 (8.5)
In non-accidental injuries, bruises are usually seen on the skin covering bone protrusions such as the forehead, shoulder, elbow and knee.*	No.	51 (35.9)
	Not sure	26 (18.3)
	Yes	65 (45.8)
The frequency of injuries and wounds in different stages of healing on the child’s body suggests the possibility of abuse.	No.	5 (3.5)
	Not sure	5 (3.5)
	Yes	132 (93.0)
Lacerations of the upper labial frenulum and bruising of the inner upper lip in children under one year of age indicate the possibility of abuse.	No.	6 (4.2)
	Not sure	26 (18.3)
	Yes	110 (77.5)
Burns are often in the form of hot object injury.	No.	14 (9.9)
	Not sure	37 (26.1)
	Yes	91 (64.1)
Repeated burns should suggest physical abuse.	No.	2 (1.4)
	Not sure	5 (3.5)
	Yes	135 (95.1)
Bite marks observed on the head and neck during dental examination/treatment are not a sign of abuse.*	No.	25 (17.6)
	Not sure	5 (3.5)
	Yes	112 (78.9)

Values are expressed as n (%) or Median [Min–Max].

In scale scoring, questions marked with an asterisk (

* ) were reverse coded. The coding scheme for the answer options is as follows: 0 for “No,” 1 for “Not sure” and 2 for “Yes.”

The participants indicated that they had encountered 2 cases (median) of child abuse during their professional life. Regarding the procedures to be followed in such cases, 79 (55.6%) of the participants stated that they had sufficient information, 45 (31.7%) that they had no information and 18 (12.7%) that they had insufficient information. 137 participants (96.5%) stated that child abuse should be included in professional training curricula, while 128 participants (90.1%) stated that they would like to receive more training on child abuse.

No significant correlation was found between the participants’ level of knowledge and skills in recognizing cases of child abuse and neglect and their perceived level of competence in identifying and managing cases of child abuse and neglect (*P* = .604). Similarly, no significant correlation was found between the level of knowledge and skills in recognizing cases of child abuse and neglect and the level of willingness to deal with cases of child abuse and neglect (*P* = .872). On the other hand, a moderate and positive significant relationship was found between the perceived level of competence in identifying and managing cases of child abuse and neglect and the level of willingness to deal with cases of child abuse and neglect (*P* < .001) (Table 3).

**Table 3:** Knowledge-skill levels in recognizing child abuse, perception of competence in recognizing abuse cases and volunteerism levels in being involved in the processes of managing abuse cases.

		*r*	*P* *
Recognition Skills Score	To what extent do you feel competent in recognizing and managing child abuse?	−0.044	.604
Recognition Skills Score	To what extent do you intend to be involved in processes to manage child abuse?	0.014	.872
To what extent do you feel competent in recognizing and managing child abuse?	To what extent do you intend to be involved in processes to manage child abuse?	0.336	**<.001**

Bold values represent statistically significant results (*P* < 0.05).

* Spearman’s rho correlation coefficient was used.

Male participants had significantly higher knowledge and skills in recognizing cases of child abuse and neglect and a perceived level of competence in identifying and managing cases of child abuse and neglect than female participants (*P* = .020 and *P* = .010, respectively). The level of knowledge and skills in identifying cases of child abuse and neglect and the perceived level of competence in identifying and managing cases of child abuse and neglect were significantly lower in pedodontists than in pediatricians (*P* = .029 and *P* < .001, respectively). The perceived level of competence in identifying and managing cases of child abuse and neglect in resident physicians was comparable to academicians but lower than specialist physicians (*P* = .099 and *P* = .024, respectively). On the other hand, there was no significant difference between specialist physicians and academicians in perceived level of competence in identifying and managing cases of child abuse and neglect (*P* = .805) (Table 4).

**Table 4:** Comparison of participants’ knowledge and skill levels in recognizing child abuse cases, their perceptions of competence in recognizing abuse cases and their level of voluntary involvement in the processes of managing abuse cases according to demographic and professional characteristics.

	Child abuse recognition knowledge and skills score §	*P*	Perception of competence in recognizing and managing child abuse (score between 0–10) §	*P*	Voluntariness in managing child abuse (score between 0–10) §	*P*
Age						
<44 years (n = 134)	23.0 [16.0–28.0]	.353*	7 [3–10]	.099*	8 [1–10]	.127*
>44 years (n = 8)	22.5 [14.0–25.0]		8 [6–9]		9.5 [6–10]	
Gender						
Female (n = 107)	24.0 [14.0–28.0]	.020*	7 [3–10]	.010*	8 [2–10]	.394*
Male (n = 35)	22.0 [19.0–28.0]		7 [4–10]		8 [1–10]	
Institution						
State Hospitals (n = 123)	23.0 [14.0–28.0]	.463*	7 [3–10]	.789*	8 [1–10]	.612*
Private Hospitals/Medical practices (n = 19)	23.0 [18.0–26.0]		7 [3–10]		8 [4–10]	
Branch						
Pediatrics (n = 53)	22.0 [18.0–28.0]	.029*	7 [3–10]	<.001*	8 [1–10]	.180*
Pedodontics (n = 89)	24.0 [14.0–28.0]		7 [3–10]		8 [3–10]	
Title						
Resident Physician (n = 63)	23.0 [16.0–28.0]		6.0 [3.0–10.0]		8.0 [3.0–10.0]	
Specialist Physician (n = 52)	23.0 [18.0–28.0]	.729†	7.0 [3.0–10.0]	.015†	8.0 [1.0–10.0]	.786†
Academician (n = 27)	24.0 [14.0–28.0]		7.0 [4.0–10.0]		8.0 [3.0–10.0]	

Values are expressed as Median [Min–Max].

* Mann–Whitney *U* test.

† Kruskal Wallis *H* test.

Participants aged 44 years and younger were less knowledgeable about protocols for reporting cases of child abuse and neglect compared to those aged over 44 years (*P* = .010). Female participants were less knowledgeable about protocols for reporting child abuse and neglect than male participants (*P* = .008). Similarly, significantly more female participants were unaware that reporting child abuse and neglect is mandatory (*P* = .028). At the same time, they were also more concerned that children who were victims of abuse and neglect would be negatively affected by the reporting of child abuse and neglect (*P* = .041). Participants working as resident physicians were significantly more concerned that children who were victims of abuse and neglect would be negatively affected by the reporting of child abuse and neglect compared to specialist physicians (*P* = .042). Participants working as resident physicians were also significantly more concerned that their professional career would be harmed by reporting of child abuse and neglect compared to specialist physicians and academicians (*P* = .036).

It was determined that pedodontists were less knowledgeable about protocols on re-porting cases of child abuse and neglect (*P* = .032), unaware that reporting child abuse and neglect is mandatory (*P* = .017), more concerned that children who were victims of abuse and neglect would be negatively affected from the reporting of child abuse and neglect (*P* < .001), and felt more incompetent in identifying cases of child abuse and neglect (*P* = .011), and needed more time to complete the procedures needed to report child abuse and neglect cases than pediatricians (*P* = .028) (Table 6). The univariate analyses revealed that the level of perceived competence in identifying child abuse and neglect cases increased by 0.94 units for male participants (*P* = .004), decreased by 1.04 units for pediatricians (*P* < .001), increased by 0.81 units for specialist physicians (*P* = .011), and 0.77 units for academicians (*P* = .048), increased by 0.29 units (*P* < .001), increased by 1.26 units for those who are not sure about receiving training on child abuse and neglect com-pared to those who want to receive training on child abuse and neglect (*P* = .025), in-creased by 0.76 units for those who stated not to have enough time to deal with child abuse and neglect cases (0.036), and decreased by 1.27 units for those who have doubts about their ability to recognize child abuse and neglect cases (*P* < .001) (Table 5).

**Table 5:** Comparison of the barriers experienced in the management of abuse cases according to demographic and professional characteristics.

		Age groups		*P* *	Gender		*P* *	Employment sector		*P* *	Specialization		*P* *	Title			*P* *
		≤44 Age (n = 134)	>44 Age (n = 8)		Female (n = 107)	Male (n = 35)		Public (n = 123)	Private (n = 19)		Pediatrics (n = 53)	Pedodontics (n = 89)		Resident Physician (n = 63)	Specialist Physician (n = 52)	Academician (n = 27)	
Fear of family/parental reaction.	Yes	74 (55.2)	4 (50)	.999	61 (57)	17 (48.6)	.500	69 (56.1)	9 (47.4)	.643	26 (49.1)	52 (58.4)	.362	38 (60.3)	27 (51.9)	13 (48.1)	.489
	No	60 (44.8)	4 (50)		46 (43)	18 (51.4)		54 (43.9)	10 (52.6)		27 (50.9)	37 (41.6)		25 (39.7)	25 (48.1)	14 (51.9)	
Lack of knowledge about the notification protocol.	Yes	81 (60.4)	1 (12.5)	.010	69 (64.5)	13 (37.1)	.008	67 (54.5)	15 (78.9)	.078	24 (45.3)	58 (65.2)	.032	39 (61.9)	33 (63.5)	10 (37.0)	.053
	No	53 (39.6)	7 (87.5)		38 (35.5)	22 (62.9)		56 (45.5)	4 (21.1)		29 (54.7)	31 (34.8)		24 (38.1)	19 (36.5)	17 (63.0)	
Not enough time to report.	Yes	27 (20.1)	1 (12.5)	.999	19 (17.8)	9 (25.7)	.434	24 (19.5)	4 (21.1)	.999	16 (30.2)	12 (13.5)	.028	11 (17.5)	14 (26.9)	3 (11.1)	.205
	No	107 (79.9)	7 (87.5)		88 (82.2)	26 (74.3)		99 (80.5)	15 (78.9)		37 (69.8)	77 (86.5)		52 (82.5)	38 (73.1)	24 (88.9)	
Worry about further harm to the child.	Yes	67 (50)	5 (62.5)	.719	60 (56.1)	12 (34.3)	.041	65 (52.8)	7 (36.8)	.293	15 (28.3)	57 (64)	<.001	39 (61.9)	20 (38.5)	13 (48.1)	.042
	No	67 (50)	3 (37.5)		47 (43.9)	23 (65.7)		58 (47.2)	12 (63.2)		38 (71.7)	32 (36)		24 (38.1)	32 (61.5)	14 (51.9)	
Worry about damage to your professional life.	Yes	41 (30.6)	2 (25)	.999	33 (30.8)	10 (28.6)	.967	39 (31.7)	4 (21.1)	.501	15 (28.3)	28 (31.5)	.836	26 (41.3)	12 (23.1)	5 (18.5)	.036
	No	93 (69.4)	6 (75)		74 (69.2)	25 (71.4)		84 (68.3)	15 (78.9)		38 (71.7)	61 (68.5)		37 (58.7)	40 (76.9)	22 (81.5)	
To think that social services cannot be the solution to this situation.	Yes	53 (39.6)	1 (12.5)	.156	42 (39.3)	12 (34.3)	.745	46 (37.4)	8 (42.1)	.889	17 (32.1)	37 (41.6)	.343	25 (39.7)	21 (40.4)	8 (29.6)	.605
	No	81 (60.4)	7 (87.5)		65 (60.7)	23 (65.7)		77 (62.6)	11 (57.9)		36 (67.9)	52 (58.4)		38 (60.3)	31 (59.6)	19 (70.4)	
Suspicion and inability to diagnose.	Yes	83 (61.9)	2 (25)	.060	68 (63.6)	17 (48.6)	.170	74 (60.2)	11 (57.9)	.999	24 (45.3)	61 (68.5)	.011	44 (69.8)	28 (53.8)	13 (48.1)	.085
	No	51 (38.1)	6 (75)		39 (36.4)	18 (51.4)		49 (39.8)	8 (42.1)		29 (54.7)	28 (31.5)		19 (30.2)	24 (46.2)	14 (51.9)	
Not wanting to get involved in legal processes.	Yes	58 (43.3)	4 (50)	.729	46 (43)	16 (45.7)	.932	55 (44.7)	7 (36.8)	.692	27 (50.9)	35 (39.3)	.240	24 (38.1)	26 (50.0)	12 (44.4)	.438
	No	76 (56.7)	4 (50)		61 (57)	19 (54.3)		68 (55.3)	12 (63.2)		26 (49.1)	54 (60.7)		39 (61.9)	26 (50.0)	15 (55.6)	
Not knowing that there is an obligation to report.	Yes	22 (16.4)	1 (12.5)	.999	22 (20.6)	1 (2.9)	.028	20 (16.3)	3 (15.8)	.999	3 (5.7)	20 (22.5)	.017	8 (12.7)	9 (17.3)	6 (22.2)	.536
	No	112 (83.6)	7 (87.5)		85 (79.4)	34 (97.1)		103 (83.7)	16 (84.2)		50 (94.3)	69 (77.5)		55 (87.3)	43 (82.7)	21 (77.8)	

Values are expressed as n (%).

* Pearson Chi-Square/Fisher’s Exact test.

**Table 6:** Factors associated with perceived levels of competence in recognizing child abuse symptoms: univariate and multiple linear regression analysis results.

	Univariate linear regression		Multivariate linear regression	
	Unadjusted coefficient [95% CI]	Unadjusted *P* value	Adjusted coefficient [95% CI]	Adjusted *P* value
Age group:*>44 Years vs <44 Years*	0.93 [−0.29 to 2.15]	.136		
Gender: *Male vs Female*	0.94 [0.3 to 1.58]	**.004**	0.57 [−0.06 to 1.2]	.081
Branch *Pedodontics vs Pediatrics*	−1.04 [−1.6 to −0.48]	**<.001**	−0.47 [−1.11 to 0.17]	.149
Employment Sector: *Private Hospitals/Ministries vs Public Hospitals*	−0.01 [−0.84 to 0.82]	.979		
Title: *ref.= Research Staff*				
*Specialist*	0.81 [0.2 to 1.43]	**.011**	0.28 [−0.29 to 0.84]	.336
*Academic Titles*	0.77 [0.01 to 1.53]	**.048**	0.38 [−0.29 to 1.05]	.263
To what extent do you intend to be involved as a volunteer in managing cases of child abuse?	0.29 [0.17 to 0.41]	**<.001**	0.3 [0.19 to 0.41]	**<.001**
Would you like to receive more training on child abuse*. = Yes*				
*No.*	0.61 [−1.08 to 2.3]	.480	0.33 [−1.13 to 1.79]	.659
*I am not sure*	1.26 [0.17 to 2.35]	**.025**	1.1 [0.13 to 2.06]	**.028**
Do you think that mechanisms for identifying and reporting possible cases of child abuse/neglect should be part of vocational training curricular*. = Yes*				
*No.*	1.27 [−2.12 to 4.66]	.464		
*I am not sure*	0.27 [−1.44 to 1.98]	.758		
Recognition Skills Score	−0.02 [−0.13 to 0.1]	.794		
Fear of family/parental reaction: *Yes vs No*	−0.18 [−0.75 to 0.39]	.542		
Lack of knowledge about the notification protocol: *Yes vs No*	−0.53 [−1.09 to 0.04]	.071		
Not having enough time to report: *Yes vs No*	0.76 [0.06 to 1.46]	**.035**	0.35 [−0.26 to 0.96]	.261
Worry about further harm to the child: *Yes vs No*	0.15 [−0.42 to 0.71]	.609		
Worry about damaging your professional career: *Yes vs No*	0.1 [−0.52 to 0.71]	.759		
To thinking that social services cannot be a solution to this situation: *Yes vs No*	0.26 [−0.32 to 0.84]	.383		
Suspicion and inability to make a diagnosis: *Yes vs No*	−1.27 [−1.81 to −0.74]	**<.001**	−0.88 [−1.37 to −0.38]	**<.001**
Not wanting to be involved in legal processes: *Yes vs No*	0.42 [−0.15 to 0.99]	.147		
Not knowing that there is an obligation to report: *Yes vs No*	−0.27 [−1.04 to 0.5]	.494		

Bold values represent statistically significant results (*P* < 0.05).

The multivariate analyses revealed that when the level of willingness in managing cases of child abuse and neglect increased by 1 unit, the perceived level of competence in identifying cases of child abuse and neglect increased by 0.3 units (*P* < .001), increased by 1.1 units among those who are not sure about receiving training on child abuse and neglect compared to those who want to receive training (*P* = .028), and decreased by 0.88 units among those who have doubts about their ability to recognize cases of child abuse and neglect (*P* < .001) (Table 6).

According to univariate analyses, the level of knowledge and skills in recognizing cases of child abuse and neglect decreased by 2.74 units in those who did not want to receive training on child abuse and neglect compared to those who wanted to receive training (*P* = .030), by 1.18 units in those who stated not to have enough time to deal with cases of child abuse and neglect (0.024), and by 6.34 units in those who are not sure about receiving training on child abuse and neglect compared to those who wanted training on child abuse and neglect to be a part of specialization training (*P* < .001). According to multivariate analyses, the level of knowledge and ability to recognize cases of child abuse and neglect decreased by 1.08 units (0.026) among those who stated not to have enough time to deal with cases of child abuse and neglect and by 6.33 units among those who are not sure about receiving training on child abuse and neglect compared to those who wanted training on child abuse and neglect to be a part of specialization training (*P* < .001) (Table 7).

**Table 7:** Factors associated with abuse recognition knowledge skill levels: univariate and multiple linear regression analysis results.

	Univariate linear regression		Multivariate linear regression	
	Unadjusted coefficient [95% CI]	Unadjusted *P* value	Adjusted coefficient [95% CI]	Adjusted *P* value
Age group:*>44 years vs <44 years*	−1.49 [−3.25 to 0.27]	.098		
Gender: *Male vs Female*	−0.86 [−1.8 to 0.08]	.073		
Branch *Pedodontics vs Pediatrics*	0.76 [−0.08 to 1.59]	.079		
Employment Sector: *Private Hospitals/Ministries vs Public Hospitals*	−0.5 [−1.7 to 0.7]	.414		
Title: *ref.= Research Staff*				
*Specialist*	0.03 [−0.89 to 0.94]	.953		
*Academic Titles*	0.22 [−0.91 to 1.34]	.706		
To what extent do you intend to be involved as a volunteer in managing cases of child abuse?	0.04 [−0.15 to 0.23]	.691		
Would you like to receive more training on child abuse*. = Yes*				
*No.*	−2.74 [−5.19 to −0.3]	**.030**	−1.41 [−3.7 to 0.89]	.231
*I am not sure*	−0.09 [−1.67 to 1.49]	.909	1.47 [−0.05 to 2.99]	.06
Do you think that mechanisms for identifying and reporting possible cases of child abuse/neglect should be part of vocational training curricular*. = Yes*				
*No.*	−0.09 [−4.54 to 4.36]	.969	−0.24 [−4.6 to 4.13]	.915
*I am not sure*	−6.34 [−8.59 to −4.09]	**< .001**	−6.33 [−8.72 to −3.94]	**< .001**
Fear of family/parental reaction: *Yes vs No*	−0.62 [−1.44 to 0.19]	.138		
Lack of knowledge about the notification protocol: *Yes vs No*	0.62 [−0.2 to 1.44]	.141		
Not having enough time to report: *Yes vs No*	−1.18 [−2.19 to −0.17]	**.024**	−1.08 [−2.03 to −0.14]	**.026**
Worry about further harm to the child: *Yes vs No*	0.72 [−0.09 to 1.53]	.083		
Worry about damaging your professional career: *Yes vs No*	−0.57 [−1.46 to 0.32]	.210		
To thinking that social services cannot be a solution to this situation: *Yes vs No*	−0.15 [−0.99 to 0.69]	.726		
Suspicion and inability to make a diagnosis: *Yes vs No*	0.52 [−0.31 to 1.35]	.221		
Not wanting to be involved in legal processes: *Yes vs No*	−0.55 [−1.37 to 0.27]	.189		
Not knowing that there is an obligation to report: *Yes vs No*	0.11 [−1 to 1.22]	.848		

Bold values represent statistically significant results (*P* < 0.05).

Multivariate analyses revealed that willingness in managing child abuse and neglect cases decreased only in participants with “fear of family/parental reaction” by 0.76 units (*P* = .043) (Table 8).

**Table 8:** Factors associated with levels of voluntary involvement in managing abuse cases: univariate and multiple linear regression analysis results.

	Univariate linear regression		Multivariate linear regression	
	Unadjusted coefficient [95% CI]	Unadjusted *P* value	Adjusted coefficient [95% CI]	Adjusted *P* value
Age group:*>44 years vs <44 years*	1.2 [−0.35 to 2.74]	.132	1.18 [−0.37 to 2.73]	.139
Gender: *Male vs Female*	−0.23 [−1.07 to 0.6]	.586		
Branch *Pedodontics vs Pediatrics*	0.41 [−0.33 to 1.15]	.282		
Employment Sector: *Private Hospitals/Ministries vs Public Hospitals*	−0.13 [−1.19 to 0.92]	.806		
Title: *ref.= Research Staff*				
*Specialist*	0.04 [−0.76 to 0.85]	.913		
*Academic Titles*	0.33 [−0.66 to 1.32]	.510		
Would you like to receive more training on child abuse* = Yes*				
*No.*	−0.33 [−2.5 to 1.84]	.767	−1 [−3.19 to 1.2]	.376
*I am not sure*	−1.03 [−2.43 to 0.38]	.153	−1.1 [−2.49 to 0.29]	.124
Do you think that mechanisms for identifying and reporting possible cases of child abuse/neglect should be part of vocational training curricula: *ref.= Yes*				
*No.*	2.24 [−2.05 to 6.53]	.308		
*I am not sure*	−1.01 [−3.18 to 1.16]	.363		
Recognition Skills Score	0.03 [−0.12 to 0.18]	.691		
Fear of family/parental reaction: *Yes vs No*	−0.66 [−1.38 to 0.05]	.072	−0.76 [−1.48 to −0.03]	**.043**
Lack of knowledge about the notification protocol: *Yes vs No*	−0.06 [−0.79 to 0.67]	.864		
Not having enough time to report: *Yes vs No*	0.23 [−0.68 to 1.13]	.624		
Worry about further harm to the child: *Yes vs No*	−0.05 [−0.77 to 0.67]	.894		
Worry about damaging your professional career: *Yes vs No*	0 [−0.79 to 0.78]	.993		
To thinking that social services cannot be a solution to this situation: *Yes vs No*	0.26 [−0.48 to 1]	.493		
Suspicion and inability to make a diagnosis: *Yes vs No*	−0.39 [−1.13 to 0.34]	.293		
Not wanting to be involved in legal processes: *Yes vs No*	−0.09 [−0.82 to 0.63]	.800		
Not knowing that there is an obligation to report: *Yes vs No*	−0.32 [−1.3 to 0.66]	.521		

Bold values represent statistically significant results (*P* < 0.05).

## 4. Discussion

This study was carried out to investigate the level of knowledge and skills, the perceived level of competence, and the willingness to identify and manage cases of child abuse and neglect in pediatricians and pedodontists who have completed or are continuing their residency training in Turkey. Consequently, pediatricians’ and pedodontists’ knowledge and skills in identifying cases of child abuse and neglect were 23/30 points, somewhat superior to the relevant results reported in the literature.^[11,12]^ Recognizing such cases can be achieved primarily through awareness. Then, knowledge and the ability to put that knowledge into practice come to the fore. WHO lists the responsibilities of healthcare professionals regarding cases of child abuse and neglect as identifying the cases of child abuse and neglect, protecting children victims of child abuse and neglect through a holistic approach and interdisciplinary cooperation, and providing appropriate treatment conditions for these children.^[18]^ Physical abuse may also occur unexpectedly during a routine physical examination, e.g., multiple and diverse healing and healed skin burns, traumatic tooth loss, oral lacerations, or an examination performed for another purpose.^[14]^

A study conducted in the Netherlands reported the prevalence of child abuse and neglect cases based on child abuse reports and self-report studies as around 3% and 12%, respectively.^[19,20]^ The prevalence of child abuse and neglect cases in our country has been reported up to 70% in different studies.^[21,22]^ In comparison, in this study, the rate of participants who stated they had encountered at least one case of child abuse and neglect was 41.5%. It does not seem very likely that pediatricians or pedodontists have not encountered a single case of child abuse and neglect. Therefore, it might be possible that the study participants who stated not to have encountered single child abuse and neglect missed these cases. One of the reasons for the low rate of participants who stated to have encountered child abuse and neglect cases despite the high prevalence of child abuse and neglect in Turkey might be that resident physicians made up a significant proportion (44.4%) of the study sample, while another reason might be that abused and neglected children rarely apply to hospitals unless there is an emergency.

Age is a noteworthy variable in identifying cases of child abuse and neglect. Healthcare professionals’ knowledge, skills, and experience significantly increase over time. Therefore, it can be expected that the likelihood of recognizing child abuse and neglect cases increases with age. Around 44% of the physicians under the age of 44 stated that they had encountered at least one case of child abuse and neglect, and all physicians over 44 stated to have encountered at least one case of child abuse and neglect. At the same time, however, our results show that more physicians over 44 were unaware that reporting child abuse and neglect is mandatory. On the one hand, these findings may indicate that awareness, knowledge, and skills training regarding child abuse and neglect might not have been covered adequately in the training of older physicians compared to younger physicians, but it may also indicate that the current training levels are still not at the expected level. Nevertheless, a study conducted in Iran showed that physicians’ knowledge of child abuse and neglect was not affected by their length of service since graduation and, to some extent, by age.^[23]^ Dalledone et al^[24]^ found that the rate of physicians who stated to have encountered a case they suspected of child abuse and neglect was higher among participants with more than 20 years of professional experience.

Encounters with child abuse and neglect cases showed a similar distribution across genders and specializations investigated in this study. Accordingly, more pedodontists stated to have encountered a case they suspected of child abuse and neglect than pediatricians, while their knowledge and skills in recognizing cases of child abuse and neglect, as well as their perceived level of competence in recognizing and managing cases of child abuse or neglect, were lower than pediatricians. These results can be interpreted as pedodontists having more difficulty than pediatricians in identifying child abuse and neglect cases. One of the main reasons for the low rate of participants who stated to have encountered child abuse and neglect may be that child abuse and neglect is not sufficiently covered in the curricula of pedodontists and pediatricians due to time constraints. Previous studies have reported the level of awareness on recognizing and dealing with cases of child abuse and neglect among healthcare professionals other than physicians between 43.8% and 60%.^[25,26]^ Pediatricians receive theoretical and practical training also in psychiatry, behavioral sciences, deontology, and forensic medicine as part of their medical education. Although pedodontists also receive training on similar subjects, the adequacy, diversity, and applicability of this training may differ from pediatricians.

This study’s findings revealed that pedodontists were less knowledgeable about protocols for reporting cases of child abuse and neglect, were more unaware that reporting child abuse and neglect is mandatory, were more concerned that children who were victims of abuse and neglect would be negatively affected from the reporting of child abuse and neglect, and had a lower level of perceived competence in identifying and managing child abuse and neglect cases. A study conducted by Türker in 2017 determined that healthcare professionals found dealing with legal procedures related to child abuse and neglect cases the most challenging aspect of managing child abuse and neglect cases.^[27]^ A study conducted in India in 2015 found that most medical and dental professionals received no training on child abuse and neglect during their undergraduate education.^[28]^Manea et al^[29]^ found that the ability of healthcare professionals to answer questions on child abuse correctly and neglect increased up to 15-fold with the widespread introduction of child abuse courses into healthcare education curricula at universities.

The gender of the participants also seemed to be an important factor in dealing with child abuse and neglect cases. According to our results, the rate of female participants who stated to have encountered a case they suspected of child abuse or neglect was higher, whereas their level of knowledge and skills and perceived competence in identifying and managing cases of child abuse and neglect were lower than male participants. This may be due to differences between genders in decision-making. Studies show that men try to solve problems quickly and analytically; thus, their solution repertoire is narrower, whereas women prefer to think more contextually and evaluate more alternatives.^[30]^ “Not being sure” of a child abuse or neglect case is common across professions and disciplines, as it is difficult to recognize cases of child abuse and neglect. Depending on their age, children may find it difficult to express what they have experienced; they may be afraid of the perpetrator or the doctor. In addition, given that the perpetrators are often relatives of the children who are victims of abuse and neglect, the children may be afraid of being harmed by the perpetrators and prefer to hide what has happened. Hence, it is particularly important in such suspicious cases to be careful to interview the child with a social worker or child psychiatrist, if possible, to carry out the examination and take the necessary steps until the suspicion is cleared up.^[31]^

Male participants stated that they were not knowledgeable about the protocols for reporting child abuse and neglect cases and that they were unaware that reporting child abuse and neglect is mandatory. On the other hand, female participants were more concerned that children who were victims of abuse and neglect would be negatively affected by reporting child abuse and neglect than male participants. Therefore, it can be speculated that male participants are more concerned with the technical aspects of child abuse and neglect cases, whereas female participants are more concerned with their emotional aspects.

The perceived level of competence in identifying and managing cases of child abuse was higher in specialist physicians than in resident physicians and academicians. This finding indicates the importance of not only knowledge but also clinical experience in both medicine and dentistry. Further analyses revealed that, as with specialist physicians, being an academician also positively affected the perceived competence level in identifying and managing cases of child abuse and neglect compared to the resident physicians. In sum, as the level of professional experience and knowledge increases, so does the perceived level of competence.^[32]^ The correlation analyses indicated that the level of knowledge and skills specific to child abuse and neglect is not related to the perceived competence in identifying and managing child abuse and neglect cases. Therefore, it can be said that the general knowledge and skills of specialist physicians and academicians positively affect their perceived level of competence in identifying and managing cases of child abuse and neglect. Another finding supporting this interpretation was that the perceived level of competence was significantly higher among participants who “did not have enough time to report,” i.e., who worked longer and more intensively. On the other hand, not having enough time to report seemed to negatively affect the knowledge and skills required for identifying and managing cases of child abuse and neglect. These findings indicate the importance of the balance between experience and knowledge in identifying and managing child abuse and neglect cases.

Another important finding was that there was no significant correlation between the level of knowledge and skills required for identifying and managing cases of child abuse and neglect and the perceived level of competence in identifying and managing cases of child abuse and neglect and the level of willingness to identify and manage cases of child abuse and neglect. It would be expected that as the level of knowledge and skills required for identifying and managing cases of child abuse and neglect increase, the perceived level of competence will also increase. However, the fact that the said positive correlation was not observed in this study may be related to the psychological characteristics of the participants, including their anxiety and depression levels and personality traits and external factors, including legislation and barriers to reporting. According to our findings, there is also a significant and positive relationship between the perceived competence in identifying and managing cases of child abuse and neglect and the willingness to identify and manage cases of child abuse and neglect. This result indicates that training alone, which increases the participants’ scientific or legal knowledge in combating child abuse and neglect, is insufficient and that psychological support and legal guarantees should also be provided to healthcare professionals.^[33,34]^

When we analyzed the factors influencing the level of willingness in identifying and managing cases of child abuse and neglect using regression models, we found that the level of willingness was negatively influenced only by “fear of family/parental reaction.” Child abuse and neglect cases are clinical cases with a forensic dimension as they impose responsibilities on healthcare professionals and the family. Family members, especially parents, are sometimes the perpetrators of abuse or neglect, and they have a duty to protect the child even if they are not perpetrators. In other words, in either case, they will face legal proceedings in cases of abuse or neglect. This situation can trigger emotions such as fear, anxiety, sadness, guilt, and aggression in parents, which may be directed toward the physician. In such cases, the clinician may feel under pressure and need support to manage the process appropriately.^[35]^

Unlike other studies, this study assessed the child abuse and neglect phenomena based on concepts such as knowledge, competence, and willingness. The results of our study, which can be generalized to larger populations due to the sampling method used, revealed the individual needs of healthcare professionals in identifying and managing cases of child abuse and neglect.

The questionnaire used to collect data was developed meticulously in several stages to achieve the desired validity and reliability. However, potential measurement errors may still stand out as the study’s primary limitation. Conducting studies on child abuse and neglect is challenging. The participants were informed about the study and assured about the anonymity of the results before the study. Nevertheless, given the ethical, emotional, and legal dimensions of these phenomena, it is still possible that the participants answered the questions “not completely spontaneously,” affecting the results. The sample size may be considered another limitation. There is no precise method for calculating the required sample size in survey studies. However, the results of our post hoc power analysis, conducted with an alpha level of 0.05, indicated a statistical power of approximately 1, which exceeds the conventional target of 0.8, thereby confirming our sample size’s adequacy for detecting medium-sized effects.

## 5. Conclusions

Find below the results of our research, which we have conducted with great care and attention to detail;

1. The importance of training and experience in acquiring the level of knowledge and skill required for identifying and managing cases of child abuse and neglect was demonstrated,
2. Different challenges and needs of healthcare professionals in identifying and managing cases of child abuse and neglect have been demonstrated, varying according to their demographic and professional characteristics,
3. The importance of psychological and legal support, in addition to scientific knowledge and skills-based training, in equipping the healthcare professional with the desired levels of knowledge, competence, and willingness has been demonstrated.

## Author contributions

**Conceptualization:** Hazal Özer, Merve Abakli İnci.

**Data curation:** Hazal Özer, Merve Abakli İnci.

**Formal analysis:** Hazal Özer.

**Investigation:** Hazal Özer.

**Methodology:** Merve Abakli İnci.

**Supervision:** Hazal Özer.

**Validation:** Hazal Özer.

**Visualization:** Hazal Özer.

**Writing – original draft:** Hazal Özer.

**Writing – review & editing:** Merve Abakli İnci.
